# A Toolbox for Tuberculosis (TB) Diagnosis: An Indian Multicentric Study (2006–2008). Evaluation of QuantiFERON-TB Gold in Tube for TB Diagnosis

**DOI:** 10.1371/journal.pone.0073579

**Published:** 2013-09-06

**Authors:** Philippe H. Lagrange, Satheesh K. Thangaraj, Rajeshwar Dayal, Alaka Deshpande, Nirmal K. Ganguly, Enrico Girardi, Beenu Joshi, Kiran Katoch, Vishwa M. Katoch, Manoj Kumar, Vemu Lakshmi, Marc Leportier, Christophe Longuet, Subbalaxmi V. S. Malladi, Deepali Mukerjee, Deepthi Nair, Alamelu Raja, Balambal Raman, Camilla Rodrigues, Pratibha Sharma, Amit Singh, Sarman Singh, Archana Sodha, Basirudeen Syed Ahamed Kabeer, Guy Vernet, Delia Goletti

**Affiliations:** 1 Service de Microbiologie, Hôpital Saint Louis, Assistance Publique- Hôpitaux de Paris, Paris VII Denis Diderot University, Paris, France; 2 BioMérieux, Marcy-l’Etoile, France and New Delhi, India; 3 SN Medical College, Agra, India; 4 Sir J.J.Group of Govt Hosp.& Grant Medical College, Mumbai, India; 5 Indian Council of Medical Research (ICMR), New Delhi, India; 6 Department of Epidemiology and Preclinical Research, L. Spallanzani National Institute for Infectious Diseases (INMI), Rome, Italy; 7 National JALMA Institute of Leprosy & Other Mycrobacterial Diseases (ICMR), Agra, India; 8 All India Institute of Medical Sciences, New Delhi, India; 9 Nizam’s Institute of Medical Sciences, Hyderabad, India; 10 Fondation Mérieux, Lyon, France; 11 Safdarjung Hospital, New Delhi, India; 12 National Institute for Research in Tuberculosis (formerly Tuberculosis Research Center), Chetput, Chennai, India; 13 Microbiology Section, P D Hinduja Hospital & Medical Research Centre, Veer Savarkar Marg Mahim, Mumbai, India; Hopital Raymond Poincare - Universite Versailles St. Quentin, France

## Abstract

**Background:**

The aim of this multicentric prospective study in India was to assess the performance of the QuantiFERON TB-Gold in tube (QFT-GIT), Tuberculin Skin Test (TST) and microbiological results as additional tools for diagnosing active tuberculosis (TB) and latent infection (LTBI) according to Human Immunodeficiency Virus (HIV) status.

**Methods:**

Individuals with and without active TB and HIV infection were enrolled between 2006–2008. QFT-GIT and TST results were analyzed per se and in combination with microbiological data.

**Results:**

Among the 276 individuals (96 active pulmonary TB and 180 no active TB) tested by QFT-GIT, 18 indeterminate results (6.5%) were found, more significantly numerous in the HIV-infected (15/92; 16.3%) than the HIV-uninfected (3/184; 1.6%)(p<0.0001). QFT-GIT sensitivity for active TB was 82.3% and 92.9% respectively after including or excluding indeterminate results. Clinical sensitivity was significantly lower in the HIV-infected (68.4%) than the HIV-uninfected (91.4%) patients (p = 0.0059). LTBI was detected in 49.3% of subjects without active TB but varied according to TB exposure. When the TST and QFT-GIT were concomitantly performed, the respective sensitivity for active TB diagnosis was 95.0% and 85.0% in the HIV-uninfected (p = 0.60), and 66.7% and 51.5% in the HIV-infected patients (p = 0.32). QFT-GIT and TST respective specificity for active TB in the HIV-uninfected was 25.0% and 57.1% (p = 0.028), and 64.8% and 83.3% in the HIV-infected (p = 0.047). In those with active TB, QFT-GIT results were not associated with microbiological parameters (smear grade, liquid culture status, time-to-positivity of culture) or clinical suspicion of active TB score (provided by the clinicians at enrollment). Combining microbiological tests with both immunological tests significantly increased sensitivity for active TB diagnosis (p = 0.0002), especially in the HIV-infected individuals (p = 0.0016).

**Conclusion:**

QFT-GIT and TST have similar diagnostic value for active TB diagnosis. In HIV-infected patients, combining microbiological tests with both immunological tests significantly increases the sensitivity for active TB diagnosis.

## Introduction

An essential factor for controlling the spread of this disease is the ability to diagnose it in its early stages, especially in the Human Immunodeficiency Virus (HIV)-infected population. Clinical examinations, combined with direct microscopic examinations of sputum samples and cultures of bacteria (whenever achievable) still remain the traditional tools for diagnosing TB. Patients with pulmonary TB (PTB) may be smear-negative for acid-fast bacilli, and mycobacterial culture may take several weeks, therefore, diagnosis is often achieved in an advanced stage of the disease [1]. Although *in vitro* amplification of mycobacterial target DNA by PCR- based methods can provide rapid results, until recently the technology was not fully standardized. It is now suitable for routine clinical practice diagnosing PTB [2]. However, a high proportion of extrapulmonary TB and smear-negative PTB are found in HIV-infected patients who require invasive procedures to confirm a diagnosis [1].

In adult TB, old and new immunological tests, such as the one century-old tuberculin skin test (TST) and all of the new commercially available IFN-γ-release assays (IGRAs) (QuantiFERON-TB®: QF-TB or T SPOT-TB ®) are almost diagnostic adjuncts [3]. The TST is not commonly used for diagnosing active TB. IGRA sensitivity has been evaluated in active TB with the TST as a surrogate marker because there is no gold standard for latent TB infection (LTBI). The TST and QFT-GIT specificity have been evaluated in healthy individuals with low TB risk in low endemic areas [3]; however, neither test is able to differentiate active TB from LTBI [4]–[6]. Thus, their suboptimal diagnostic performances in highly endemic areas for TB suggest that IGRAs alone are not sufficient for active TB diagnosis [7].

One of the objectives of the TB control management group was to make an extensive evaluation to identify the right combination of tools for the toolbox. The first part of our multicentric prospective study in India was to assess the diagnostic value of several microbiological tools [8]. The aim of this second part was to assess the performance of the QFT-GIT and TST, in addition to microbiological results, as contributors for diagnosis of active TB according to HIV status. The specificity of both tests was assessed in healthy non-active TB individuals.

## Materials and Methods

### Study Populations

Written informed consent was obtained from all subjects before enrollment. The study was approved by the local ethical committees: the Institutional Ethical Committee of Tuberculosis Research Centre in Chennai (TRC-IEC No: 2006005), the Institutional Review Board at Hinduja Hospital, Mumbai (No: 316-05-CR), the Institutional Ethical Committee of AIIMS, New Delhi (AIIMS-IEC No: A-35∶05/10/2005), the Institutional Ethical Committee of the Safdarjung Hospital, New Delhi (Nu Sur./1/2007), the NIMS Institutional Ethics Committee (No.EC/264 (A)/2005) and the Ethical Committee of National JALMA Institute for Leprosy and other Mycobacterial Diseases, Agra (minutes: 27/04/2006).

Adult patients with active TB and individuals without active TB were prospectively enrolled from January 2006 to July 2008 at different medical centers as described [8].

In brief, clinical symptoms and radiological findings of the patients were first assessed independently by each clinician taking part in the enrollment. Three sputum samples were collected and processed. The smear was stained using the hot Ziehl-Neelsen method and the semi-quantitative yield of acid-fast-bacilli (AFB) was recorded according to WHO recommendations [9]. The 3 sputum samples were cultured in solid and liquid medium and the presence of *M.tuberculosis* in the positive culture samples was further confirmed by molecular Gen-probe based PCR.

Non-active TB individuals (negative controls) were also enrolled: blood donors, healthy community adults (HCA), healthy family contacts (HFC), health care workers (HCW-laboratory staff and nurses), and cured TB patients. Because the different settings are endemic to TB, to rule out the suspicion of active TB disease all subjects (except for blood donors) were asked to give one to three sputum samples and were subjected to radiological examinations. All enrolled healthy individuals were stratified by risk for TB exposure and were divided into “low TB risk” (blood donors and healthy community adults) and “high TB risk” (HCW, healthy family contacts, and cured TB). Subjects at high risk of TB were included on one condition: adherence to a six month follow-up to exclude the occurrence of active TB.

Each individual’s data were then recorded at each site using a standardized questionnaire involving 3 files (clinical/radiological evaluation, clinical/radiological follow-up and laboratory analysis) for patients with active TB and control groups. A clinical suspicion of TB (CSTB) score was given by the clinician in charge of patient inclusion before any microbiological and immunological results were known and each included individual was classified into 3 categories: very high, high, and low, as previously reported [8]. CSTB was used as a pre-test evaluation before enrollment. However, the study and the data analysis were performed using samples with a definite diagnosis of “active TB” or “no active TB”.

### Tuberculin Skin Test

Two TU (tuberculin units) of purified protein derivative (PPD) RT23 (Staten Serum Institute, Copenhagen, Denmark) were injected intradermally by Mantoux method and the induration was measured by trained professionals 48–72 hrs after PPD injection. An induration of ≥10 mm was considered positive at baseline, in accordance with Indian guidelines. [10].

### Interferon-gamma Release Assay (IGRA)

The specific i*n vitro* cellular immune response was evaluated using QuantiFERON TB-Gold in tube (QFT-GIT) (Cellestis, Carnegie, Australia). Blood was harvested in individuals before the TST; the QFT-GIT was then performed in the respective microbiology laboratories. The test results were interpreted using the manufacturer’s software and the cut-off point for the diagnosis determined according to the manufacturer’s instructions. Results were considered positive if the IFN-γ level in the TB (ESAT-6, CFP-10 and TB7.7) antigen-exposed sample was ≥0.35 IU.mL^−1^ after subtracting the level in the negative control (NIL) and ≥25% of the IFN-γ concentration in the NIL. Indeterminate results were defined as either an unstimulated IFN-γ level of ≥8.0 IU.mL^−1^ in the NIL plasma or an IFN-γ response of <0.5 IU.mL^−1^ on phytohaemagglutinin stimulation with a level of IFN-γ in the TB antigen-exposed sample minus the level in the NIL of either <0.35 IU.mL^1^ or <25% of the IFN- γ concentration in the NIL.

Two estimates of the QFT-GIT-positive rate were calculated: the first estimate corresponded to the calculated “clinical performance” of a biological test (i.e. number of positive tests/total number of tested patients) including the indeterminate results as negative. The second estimate corresponded to the calculated “laboratory performance” of a biological test (number of positive tests/number of interpretable results obtained) excluding the indeterminate results.

Partial results of QFT-GIT used as positive control assay for the IP10 assay have been reported in Goletti and collaborators [11]–[13]. CD4+ and CD8+ T-cell counts of HIV-infected patients were not included in the study because they were only recorded in a minority of individuals.

#### HIV serological status

HIV infection was diagnosed by two serological ELISA (Retroquic Comb Aids-RS, Span Diagnostics, India and HIV TRI-DOT, J. Mitra & Co, India). The results were scored as positive when the serum was positive by both tests. If a serum was reactive in only one ELISA, HIV-Western Blot was performed as a confirmatory test to rule out a false ELISA test result [8]. The HIV-infected subjects were not undergoing antiretroviral therapy.

### Data Collection

After collection, the data were subsequently transferred to EPIINFO files by one of the authors (ST). Each file included the patient’s characteristics (serial number, study center, date of enrollment, nature of specimen collected, patient study group, age, sex, permanent address), risk of TB, clinical symptoms (cough for more than 2 weeks, persistent low-grade fever –higher than 37°.5C for 2 weeks, weight loss -more than 10% of the usual weight within the last 3 months, night sweats, anorexia, fatigue, dyspnea, chest pain and haemoptysis), whether TST was performed or not (if performed, the diameter of induration was recorded), chest X-ray findings, the effect of a 10-day antibiotic trial, the final clinical diagnosis, CSTB, final therapeutic intervention with the therapy initiation date and the anti-TB drugs prescribed. The last part of the file consisted of treatment outcome obtained during the 6 to 9 month follow-up of each individual with TB (clinical symptoms relief, chest X-rays and microbiological conversion) and the absence of clinical symptoms in the non-active TB control groups. Patients with both pulmonary and extrapulmonary localizations (infiltrate and pleural effusion, for instance) were classified as pulmonary TB.

The presence of active pulmonary TB was defined as positive for sputum smear microscopy and finally as “microbiologically confirmed” after identification of *M. tuberculosis* in culture by the Gen-Probe (San Diego, USA) Accuprobes assay. Conversely, the patients who were smear and culture negative but had symptoms and anomalies on chest X-rays suggestive of TB and had concluded 10 days of ineffective broad spectrum antibiotics were then treated with the 4-drug anti-tuberculosis therapy (ATT) and followed up for 6 months. If the subjects responded to ATT, then they were considered as having “clinical” pulmonary TB.

### Statistical Analysis

Median and interquartile (IQR) ranges were calculated. Sensitivity and specificity were calculated as recommended [14]. Mann-Whitney U test was carried out to calculate the difference between the groups. For categorical variables Chi square was used. P values were considered significant if p≤0.05. However, for multiple comparisons, Bonferroni correction was used: for 5 multiple comparisons, p values were considered significant if p≤0.01; for 7 multiple comparisons, p values were considered significant if p≤0.007. Spearman Rank Correlation was used to correlate continuous variables.

Analysis was carried out with SPSS v 14 for Windows (SPSS Italia SRL, Bologna, Italy), GraphPad Prism 6.0 software and EPIINFO (CDC, Atlanta, USA).

## Results

### Characteristics of the Enrolled Population [8]

**Figure 1:**
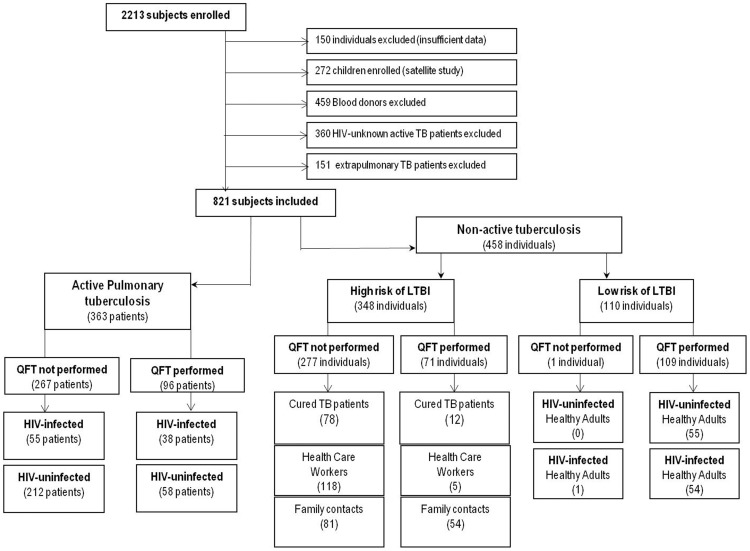
Flow chart of patients recruited to the multicentric study stratified by patient subgroups. Abbreviations: TB: tuberculosis; HIV: human immunodeficiency virus; LTBI: latent tuberculosis infection.

We enrolled 2,213 individuals in the 2-year period. We deleted 150 patient files because they were incomplete. We also deleted 459 healthy blood donors and 360 TB patients because none of them had been tested for HIV or had recorded QFT-GIT results (**Figure 1** **).** Extrapulmonary TB patients were also excluded because only very few had QFT-GIT results (7/151). Additionally, 272 children were evaluated separately [15]. The remaining files correspond to 363 active-PTB patients: 93 HIV-infected, 270 HIV-uninfected. Among the 458 adults without active TB, we identified two main groups according to their risk of TB exposure: 110 individuals [healthy community adults (HCA)] with a relatively low risk of TB infection (55 HIV-uninfected and 55 HIV-infected) and 348 with a relatively higher risk of TB infection [123 health care workers (HCW), 90 cured-TB patients, and 135 recent healthy family contacts (HFC)]. The QFT-GIT assay was only performed in a fraction of non-active TB individuals (180/441: 40.8%) and PTB patients (96/363; 26.5%), more frequently in the HIV-infected (40.9%) than in the HIV-uninfected PTB group (21.5%).

The characteristics of the PTB patients stratified by HIV status with an IGRA record are presented in **Table 1**. HIV-infected patients were more frequently male than female (p = 0.03), had less frequency of prolonged cough (p<0.0001), sputum smear (p = 0.037) culture positivity (0.009) and TST positive (0.02) results than the HIV-uninfected group. Subsequently, the very high CSTB score was significantly lower in the former than in the latter (p = 0.0001).

**Table 1:** Characteristics of the 96 Active Pulmonary TB patients included in the study.

	Active pulmonary TB patients			
	HIV-infected (N = 38)	HIV-uninfected (N = 58)	All (N = 96)	*p value HIV-infected Vs HIV-uninfected*
**Age median (IQR)**	38.0 (33.75–40.25)	37.0 (21.75–45.0)	38.0 (30.5–42.0)	*0.4288*
**Male/female**	33/5	38/20	71/25	*0.0311*
**Male prevalence (%; 95% CI))**	86.8 (71.9–95.6)	65.5 (51.9–77.5)	74.0 (64.5–82.1)	
**Main Clinical symptoms**				
** Cough (%)**	23/38 (60.5%)	58/58 (100.0%)	91/96 (94.8%)	*<0.0001*
** Weight loss (%)**	33/38 (86.8%)	50/58 (86.2%)	83/96 (86.5%)	*1.0*
** Fever (%)**	24/38 (63.2%)	33/58 (56.9%)	57/96 (59.4%)	*0.6714*
**Smear microscopy positive (%)**	19/38 (50.0%)	46/58 (79.3%)	65/96 (67.7%)	*0.037*
**Smear microscopy grade**				
** Negative (%)**	19 (50.0%)	12 (20.7%)	31 (32.3%)	*0.037*
** Positive: scanty (%)**	3 (7.9%)	6 (10.3%)	9 (9.4%)	*1.0*
** Positive:+(%)**	6 (15.8%)	12 (20.7%)	18 (18.8%)	*0.6034*
** Positive:++(%)**	8 (21.1%)	17 (29.3%)	25 (26.0%)	*0.4771*
** Positive:+++(%)**	2 (5.3%)	11 (19.0%)	13 (13.5%)	*0.0701*
**Culture positive (%)**	17/38 (44.7%)	46/58 (79.3%)	63/91 (69.2%)	*0.0018*
**TB confirmed (%)**	44.7%	79.3%	69.2%	
**Clinical TB in % (95% CI)**	55.3% (30.8–99.5)	20.7% (11.2–33.4)	30.8%	*0.0018*
**TST tested (%)**	33/38 (86.8%)	20/58 (33.3%)	53/96 (55.2%)	
**TST positive (%)**	17/33 (51.5%)	17/20 (85.0%)	34/53 (64.2%)	*0.0185*
**Median TST (IQR)**	10.0 (0–20)	16.5 (10–22)	15.0 (0–20.0)	*0.0240*
**Clinical TB suspicion (%)**				
** Very high (%)**	19 (50.0%)	51 (87.9%)	70 (72.9%)	*0.0001*
** High (%)**	19 (50.0%)	7 (12.1%)	26 (27.1%)	*0.0001*
** Low (%)**	0 (0.0%)	0 (0.0%)	0 (0.0%)	*NA*

Footnotes: TB: tuberculosis; HIV: human immunodeficiency virus; TST: tuberculin skin test with a cut-off point at 10 mm; NA: not available.

### Immunological Results

#### Cell-mediated in vitro assay (Interferon Gamma Release Assay- IGRA)

Evidence reviewed elsewhere [16], suggests that IGRAs are more specific than the TST in Bacille Calmette Guérin (BCG)-vaccinated individuals, and correlate better with markers of TB infection in low- incidence settings. The accuracy of QFT-GIT for active TB diagnosis was evaluated in PTB patients and for Latent TB infection (LTBI) diagnosis in 180 individuals with non-active TB at different risks of TB exposure.

#### Individual interferon-gamma (IFN-γ) results

**Figure 2:**
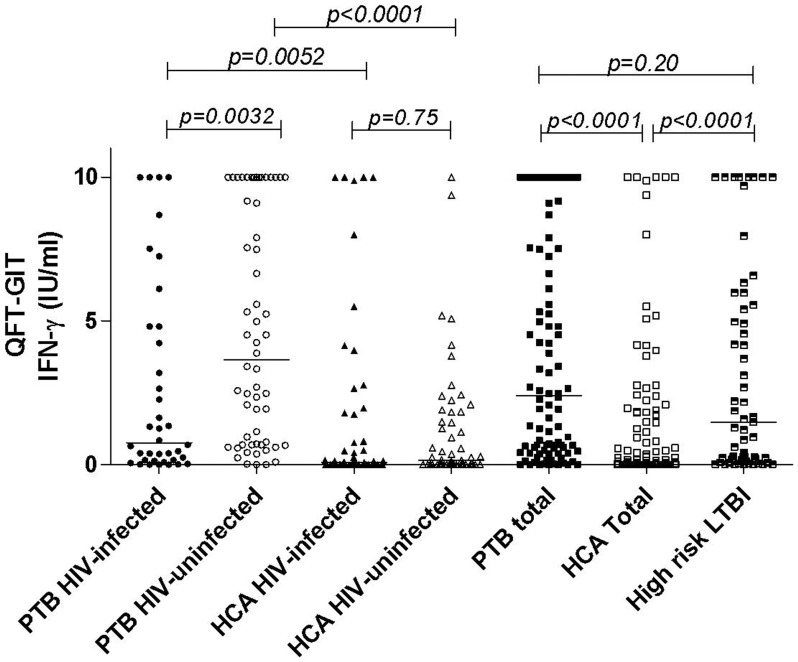
Individual Interferon-gamma (IFN-γ) responses among 96 pulmonary TB (PTB) patients and 110 healthy community adults (HCA) stratified by HIV status and in a group of 59 healthy adults at high risk of *M.tuberculosis* exposure (54 recent family contacts and 5 health care workers). Short bars, median level of QFT-GIT response in each group. Footnotes: TB: tuberculosis; HIV: human immunodeficiency virus; LTBI: latent tuberculosis infection.

The individual INF-γ level (median and IQR) was significantly higher among the whole group of PTB patients (median, 2.41, IQR 0.48–7.54 IU/ml) compared to those of HCA (median, 0.10, IQR 0.0–1.8 IU/ml) considered as a low risk of *M.tuberculosis* exposure (p<0.0001) (**Figure 2**). Among the PTB patients, the IFN-γ level was significantly lower in the HIV-infected (median, 0.77, IQR 0.15–4.81 IU/ml) than the HIV-uninfected patients (median, 3.65, IQR 0.69–10.0 IU/ml) (p = 0.0032). In contrast, among the HCA considered as low risk of *M.tuberculosis* exposure individuals, the IFN-γ level was not significantly different between the HIV-infected (median, 0.08, IQR 0.01–1.76 IU/ml) and the HIV-uninfected (median, 0.15, IQR 0.0–1.83 IU/ml) HCA (p = 0.75). The IFN-γ level was significantly higher in the group of individuals at higher risk of recent *M.tuberculosis* exposure (recent healthy family contacts and HCW) (median, 1.48, IQR 0.16–5.56 IU/ml) compared to the whole HCA group (p<0.0001) and to the HIV-uninfected HCA group (p = 0.0002), but was not significantly different than the whole PTB group (p = 0.20).

#### IGRA indeterminate results

Among the 276 individuals (96 PTB patients and 180 individuals without active TB) tested by QFT-GIT, 18 (11 with PTB and 7 without active TB) were scored indeterminate (6.5%). All the indeterminate results were due to low IFN-γ production in the positive control tube (median, 0.06, IQR 0.007–0.28). Among the PTB patients, the proportion of indeterminate results was significantly higher in the HIV-infected (9/38) than the HIV-uninfected (2/58) subjects (p = 0.006)(**Table 2** **)**. Similarly, among all the no active-TB individuals, the rate of indeterminate results was significantly higher in the HIV-infected HCA (6/54) than in the HIV-uninfected individuals (1/126) (p = 0.0032) (**Table 3**).

**Table 2:** Number and percentage (95% CI) of indeterminate results, number of positive and interpretable results and positive sensitivity rates (percentage; 95% CI) of QuantiFERON® Gold in Tube (QFT-GIT) in pulmonary TB patients stratified by HIV status and disease localization.

Active Pulmonary TB patients	HIV-infected	HIV-uninfected	Total patientstested	*p value; HIV-infected vs* *HIV-uninfected*
	(N = 38)	(N = 58)	(N = 96)	
N of indeterminate results	**9**	**2**	**11**	
Percentage of indeterminate results (95% CI)	23.7 (11.4–40.2)	3.5 (0.9–11.9)	11.5 (5.9–19.6	*0.006*
N of positive results^(a)/^N of interpretable results(b)	26/29	53/56	79/85	
Clinical sensitivity(c) (%; 95% CI)	**68.4** (51.4–82.5)	**91.4** (81.1–96.3)	**82.5 (73.2–89.3**	*0.0053*
Laboratory sensitivity(d) (%, 95% CI)	**89.7** (72.2–97.8)	**94.6** (85.1–98.2)	92.9 (83.8–96.6	*0.4058*

Footnotes: QFT-GIT: QuantiFERON® Gold in Tube; HIV: human immunodeficiency virus; TB: tuberculosis.

(a) QFT-GIT with specific antigen cut –off point: 0.35 IU/mL.

(b) QFT-GIT with PHA cut –off point: 0.50 IU/mL.

(c) Clinical sensitivity: overall sensitivity calculated on the total of patients tested (indeterminate results included as negative).

(d) Laboratory sensitivity: sensitivity calculated only on the interpretable results (indeterminate results excluded).

**Table 3:** Number and percentage (95% CI) of indeterminate results, number of positive and interpretable results and sensitivity rates (percentage; 95% CI) of QuantiFERON® Gold in Tube (QFT-GIT) in groups of individuals without active TB at varying risks of *M. tuberculosis* exposure.

Individuals at varying risks of TB exposure	Healthy community adults		Health CareWorkers	Healthy familycontacts	Cured TBpatients	Total non- activeTB subjects
	HIV-uninfected(N = 55)	HIV-infected(N = 54)	(N = 5)	(N = 54)	(N = 12)	(n = 180)
N of Indeterminate Results	0	6	0	1	0	7
Percentage of Indeterminate Results(CI 95%)	0.0 (0.0–6.8)	**11.1** (5.1–22.5)	0.0 (0.0–44.4)	1.9 (0.3–10.2)	0.0 (0.0–25.0)	3.9 (1.6–7.8)
N of positive results(a)/N of interpretable results(b)	23/55	19/48	3/5	33/53	11/12	89/173
Clinical sensitivity (%; 95% CI)(c)	**41.8** (28.7–55.9)	**35.2** (22.7–49.4) *(p = 0.5563)*	**60.0** (14.6–94.7) *(p = 0.6439)*	**61.1** (46.9–74.1) *(p = 0.0559)*	**91.7** (61.5–99.8) *(p = 0.0028)*	**49.3** (41.9–57.0)
Laboratory sensitivity (%; 95% CI)(d)	**41.8** (29.5–55.2)	**39.6** (26.8–54.0) *(p = 0.8432)*	**60.0** (14.6–94.7) *(p = 0.6439)*	**62.3** (47.9–75.2) *(p = 0.0367)*	**91.7** (61.5–99.8) *(p = 0.0028)*	**51.5** (43.7–59.1)

Footnotes: QFT-GIT: QuantiFERON® Gold in Tube; HIV: human immunodeficiency virus; TB: tuberculosis.

(a) QFT-GIT with specific antigen cut –off point: 0.35 IU/mL.

(b) QFT-GIT with PHA cut –off point: 0.50 IU/mL.

(c) Clinical sensitivity: overall sensitivity calculated on the total of patients tested (indeterminate results included as negative).

(d) Laboratory sensitivity: sensitivity calculated only on the interpretable results (indeterminate results excluded).

#### Sensitivity of QFT-GIT for active-PTB diagnosis

The results are given in **Table 2** **.** Among the whole PTB patient group, the overall QFT-GIT “clinical” sensitivity, including all indeterminate results as negative, was significantly lower (82.3%) than the “laboratory sensitivity” (92.9%) (p = 0.0431) Additionally, the “clinical sensitivity” was significantly lower in the HIV-infected (68.4%) than the HIV-uninfected (91.4%) PTB patients (p = 0.0045,). When the interpretable results were analyzed, the “laboratory sensitivity” increased significantly in the HIV-infected by 21.3% (p = 0.045) but only by 3.2% in the HIV-uninfected PTB patients (p = 0.71), subsequently, the QFT-GIT “laboratory sensitivity” was not significantly different between the HIV-infected (89.7% and HIV-uninfected (94.6%) PTB patients (p = 0.4058).

#### Sensitivity of the QFT-GIT for Latent TB Infection (LTBI) diagnosis

The results are given in **Table 3** **.** Among the healthy community adults (HCA), considered as a relatively low risk of *M. tuberculosis* exposure group, the QFT-GIT “laboratory” sensitivity was slightly lower in the HIV-infected (39.6%) than the HIV-uninfected HCA (41.8%); the difference was not significant (p = 0.84). Among the HIV-infected HCA, 6 indeterminate results (11.1%) were found; consequently the “clinical” sensitivity decreased (from 39.6% to 35.2%) compared to the “laboratory” sensitivity, but the difference between the HIV-infected and HIV-uninfected HCA was not significant (p = 0.55).

Compared to the HIV-uninfected HCA, the “clinical sensitivity” was almost significantly higher in groups with a high risk of *M. tuberculosis* exposure, such as the healthy family contacts (HFC) (61.1%) (p = 0.0559) whereas it was not significant compared to HCW (60.0%) (p = 0.64). To note: almost all cured PTB patients were QFT-GIT- positive (91.7%) 2 years after a successful treatment.

#### Diagnostic values of QFT-GIT

The sensitivity, specificity, and positive and negative likelihood ratio (LR) of the QFT-GIT assay for PTB diagnosis were calculated and the results are shown in **Table 4**. The specificity was calculated from the group of HIV-uninfected HCA as negative controls for the HIV-uninfected PTB patients and from the group of HIV-infected HCA as negative controls for the HIV-infected PTB patients (**Table 3**). The overall specificity was evaluated from the whole HCA group as negative controls for the whole group of PTB patients.

**Table 4:** Diagnostic values of QFT-GIT in active pulmonary TB patients stratified by HIV status calculated over the total of patients tested (indeterminate results included as negative results) and only over the interpretable results (indeterminate results excluded).

HIV status	Indeterminate results included as negative results				Indeterminate results excluded				
	Sensitivity	Specificity	LR+	LR−	Sensitivity	Specificity		LR+	LR−
	Percentage (95% CI)				Percentage (95% CI)				
**HIV-infected**	**68.4** (51.4–82.5)	**64.8** (50.6–77.3)	**1.95**	**2.05**	**89.7** (72.7–97.8)		**60.4** (45.3–74.2)	**2.26**	**5.86**
**HIV-uninfected**	**91.4** (81.0–97.1)	**58.2** (44.1–71.4)	**2.19**	**6.77**	**94.6** (85.1–98.9)		**58.2** (44.1–71.4)	**2.26**	**10.78**
**Total**	**82.3**(73.2–89.3)	**61.5** (51.7–70.6)	**2.13**	**3.48**	**92.9** (85.3–97.4)		**59.2** (49.1–68.8)	**2.27**	**8.34**

Footnotes: QFT-GIT: QuantiFERON® Gold in Tube; HIV: human immunodeficiency virus; TB: tuberculosis; PPV: positive predictive value; NPV: negative predictive value; LR+: positive likelihood ratio; LR.-: negative likelihood ratio.

The overall specificity was not significantly different when the indeterminate results were excluded (59.2%) or not (61.5%) (p = 0.78). The positive LR was low, with no differences between the HIV-infected and HIV-uninfected PTB patients, whereas the negative LR was higher among the HIV-uninfected compared to HIV-infected PTB patients, and increased in both groups when the indeterminate results were excluded.

#### Performance of the QFT-GIT in confirmed and clinical PTB

The varying sensitivities of the QFT-GIT assay in patients with “confirmed PTB” (culture-positive) and “clinical PTB” (smear-negative/culture-negative) are presented in **Table 5** **.** A lower sensitivity was observed in the “clinical” PTB patients than in the microbiologically confirmed PTB, but the difference was only significant among the HIV-infected PTB patients when the indeterminate results were included as negative (p = 0.0336).

**Table 5:** Sensitivity of QFT-GIT calculated over the total of patients tested (indeterminate results included as negative results) and only over the interpretable results (indeterminate results excluded) in PTB patients stratified by HIV status according to culture status.

	Indeterminate results included as negative results			Indeterminate results excluded		
	Culture-positive	Culture-negative	*p value (Culture- negative versus culture -positive)*	Culture-positive	Culture-negative	*p value (Culture-negative versus culture-positive)*
	**Positive over total % (95% CI)**					
**HIV-infected**	15/17 **88.2**(63.6–98.5)	11/21 **52.4**(29.8–74.3)		15/15 **100.0**(78.2–100)	11/14 **78.6**(49.2–95.3)	
			*0.0336*			*0.0996*
**HIV-uninfected**	42/46 **91.3**(79.2–97.6)	11/12 **91.7**(61.5–99.8)		42/44 **95.5**(84.5–99.4)	11/12 **91.7**(61.5–99.8)	
			*1.0*			*0.5222*
**Total**	57/63 90.5(80.4–96.4)	22/33 66.7(48.2–82.0)		57/59 **96.6**(88.3–99.6)	22/26 **84.6**(65.1–95.6	
			*0.009*			*0.067*

Footnotes: QFT-GIT: QuantiFERON® Gold in Tube; TB: Tuberculosis; HIV: Human Immunodeficiency Virus.

Because IGRAs and smear microscopy are both rapid tests, obtaining results in 24 h, we compared the sensitivity of QFT-GIT among smear-negative and smear-positive PTB patients. Irrespective of HIV status, no significant difference was found between the smear-negative and smear-positive PTB patients evaluated by “clinical” sensitivity **(** **Table 6**); however, the overall “laboratory” sensitivity of QFT-GIT was significantly higher in the smear-positive (98.2%) compared to the smear-negative (82.8%) (p = 0.0163) with no significant difference between the smear-negative and the smear positive in the HIV-infected (p = 0.2463) or in HIV-uninfected patients (p = 0.1127).

**Table 6:** Sensitivity of QFT-GIT calculated over the total of patients tested (indeterminate results included as negative results) and only over the interpretable results (indeterminate results excluded) in PTB patients stratified by HIV status according to smear microscopy status.

	Indeterminate results included as negative results			Indeterminate results excluded		
	AFB-positive	AFB-negative	*P (AFB- negative* *versus AFB -positive)*	AFB-positive	AFB-negative	*P (AFB- negative* *versus AFB -positive)*
	**Positive over total % (95% CI)**					
**HIV-infected**	12/19	14/19		12/12	14/17	
	**63.2** (38.4–83.4)	**73.7** (48.8–90.9	*0.7281*	**100.0** (73.5–100)	**82.4** (56.6–96.2)	*0.2463*
**HIV-uninfected**	43/46	10/12		43/44	10/12	
	**93.5** (82.1–98.6)	**83.3** (51.6–97.9)	*0.2735*	**97.7** (88.0–99.9)	**83.3** (51.6–97.9)	*0.1127*
**Total**	55/65	24/31		55/56	24/29	
	**84.6** (73.5–92.4)	**77.4** (58.8–90.4)	*0.4031*	**98.2** (90.5–99.9)	**82.8** (64.2–94.2)	*0.0163*

Footnotes: QFT-GIT: QuantiFERON® Gold in Tube; TB: Tuberculosis; HIV: Human Immunodeficiency Virus, AFB: Acid Fast Bacilli.

**Figure 3:**
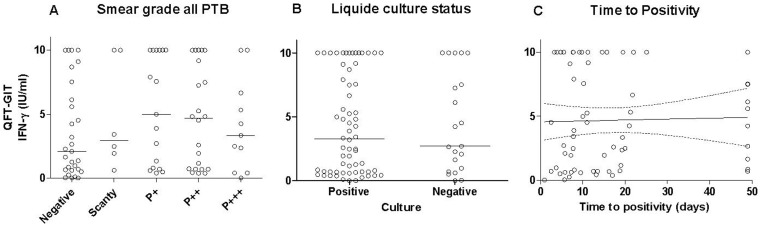
Relationship between individual quantitative IFN-γ results (IU/ml) and the sputum bacillary load expressed as (A) smear grade (p = 0.64), (B) liquid culture results (p = 0.83), and (C) time-to-positivity (TTP) of the liquid culture (R^2^ = 0.00082, p = 0.82), measured in the active pulmonary tuberculosis patients with interpretable IFN-γ results (indeterminate excluded). Short bars, median level of QFT-GIT response in each group. Abbreviations: IFN-γ: interferon-γ.

Moreover, such independence between the sensitivity of the QFT-GIT assay and smear status was also verified assessing the relationship between the sputum bacterial load and the quantitative QFT-GIT results. The IFN-γ level was not significantly associated with the bacillary load expressed as the smear grade (p = 0.32), liquid culture positivity (p = 0.92) or liquid culture time-to-positivity (TTP) (**Figure 3** **)**.

#### Diagnostic values of the QFT-GIT assay according to clinical suspicion

The “clinical” sensitivity of QFT-GIT was not significantly different in patients with different CSTB scores (provided by the clinicians at enrollment) or different HIV status (**Table 7**). The pre-test evaluation using the clinical suspicion score did not significantly increase the positive (p = 1) and negative likelihood ratio (p = 0.7).

**Table 7:** Influence of the physicians’ clinical suspicion of tuberculosis (CSTB) on rate of QFT-GIT results in active pulmonary TB patients according to their disease localisation and HIV status.

HIV status	TB diagnosis*	CSTB Low	CSTB High	CSTB Very High	*p value “TB Diagnosis” vs “CSTB very high*
	**N of QFT-GIT-responders/N of patients tested (%)**				
**HIV-infected**	26/38 (68.4)	0/0 (NA)	13/19 (68.4)	13/19 (68.4)	*1.000*
**HIV-uninfected**	53/58 (91.4)	0/0 (NA)	5/7 (71.4 )	48/51 (94.1)	*0.7209*
**Total**	79/96(82.3)	0/0 (NA)	18/26 (69.2)	62/76 (81.6)	*1.000*

Footnotes: QFT-GIT: QuantiFERON® Gold in Tube; TB: tuberculosis;

* TB diagnosis by QFT-GIT (“clinical sensitivity”) within the group considered; HIV: human immunodeficiency virus; CSTB: clinical suspicion of TB; NA: not applicable.

#### Diagnostic values of QFT-GIT in comparison with TST and smear microscopy

Only a fraction (53/96: 55.2%) of the PTB patients tested by QFT-GIT had a recorded TST: 33/38 (86.8%) were HIV-infected and 20/60 (33.3%) were HIV-uninfected. Similarly, only 82/109 (75.2%) of the HCA had a recorded TST: 54/55 (98.2%) were HIV-infected and 28/54 (51.8%) were HIV-uninfected. The diagnostic values of the QFT-GIT were re-evaluated in the group of the 53 PTB patients and the 82 HCA, being concurrently tested. Assessment of the quality of the sub-grouping procedure revealed that the QFT-GIT sensitivity in TB patients with TST records was not significantly different from the overall group (p = 0.67) or those without TST records (p = 0.15), independent of HIV status.

**Figure 4:**
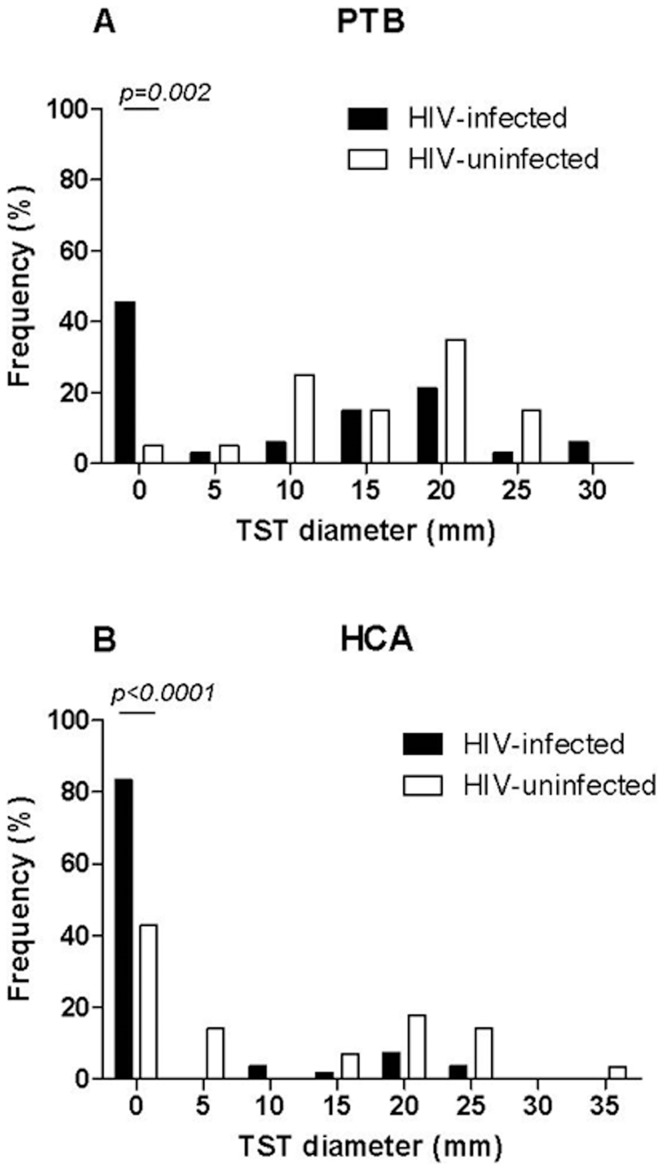
Comparative distribution of continuous TST reactions (induration in mm) in TB patients stratified by HIV status. (A) Upper graph: HIV-infected (black bar) and HIV-uninfected patients (white bar); and (B) lower graph: no active-TB individuals stratified by HIV status: HIV-infected (black bar), HIV-uninfected individuals (white bar). Abbreviations: TB: tuberculosis; HIV: human immunodeficiency virus; TST: tuberculin skin test.

The distribution of continuous TST reactions was evaluated in the PTB patients and HCA stratified by HIV status (**Figure 4**). The proportion of complete unresponsiveness (TST = 0 mm) among the PTB patients was significantly higher in the HIV-infected (46.0%; 95% CI: 29.5–63.1) than the HIV-uninfected (5%; 95% CI: 1.2–24.9) patients (p = 0.0021) and 8/9 (88.9%) of the QFT-GIT indeterminate results in the HIV-infected presented this complete TST unresponsiveness. Similarly, among the HCA the proportion of complete unresponsiveness was significantly higher in the HIV-infected (83.3%; 95% CI: 70.7–92.1) than the HIV-uninfected (33.8%; 95% CI: 22.8–46.3) (p<0.0001), and all QFT-GIT indeterminate results in the HIV-infected HCA presented this complete TST unresponsiveness. To note: only 1/37 of the HIV-infected active-TB patients had a TST of 5 mm, and none of the no active-TB individuals had a TST result between 0 and 10 mm. Therefore in this study, the diagnostic value of TST was calculated using a 10 mm cut-off point.

The overall TST sensitivity evaluated in all PTB patients was 64.2% (**Table 8**); if we stratify for HIV status, TST sensitivity was significantly lower in the HIV-infected than the HIV-uninfected patients (p = 0.0021). The overall specificity evaluated in HCA was 74.4% (**Table 8**); if we stratify for HIV status, TST specificity was significantly lower in the HIV-uninfected than the HIV-infected HCA (p<0.0001). If we compare TST and QFT-GIT sensitivity, no significant differences were found, irrespective of HIV status, although a higher sensitivity was found by QFT-GIT; however, the overall TST specificity for PTB was significantly higher than the QFT-GIT specificity when considering the overall data (p = 0.04) and results from the HIV-infected (p = 0.004) patients.

**Table 8:** Analysis of combination of the tests evaluated over 53 active pulmonary TB patients and 82 HCA being concomitantly tested and stratified by HIV status.

Positive over total tested. Percentage (95% CI)								Negative over total tested. Percentage (95% CI)						
	Sensitivity							Specificity						
	TST	QFT-IT	SM	QFT-GITwith TST	SMwith TST	SM withQFT-GIT	QFT-GITwith TSTwith SM	P Value*	TST	QFT-IT	SM	QFT-GITwith TST	QFT-GITwith TSTand SM	*p value* *
**HIV-infected**	17/33. **51.5** (33.5–69.2)	22/33. **66.7** (48.2–82.0)	15/33. **45.5** (28.1–63.7)	24/33. **72.7** (54.5–86.7)	24/33. **72.7** (54.5–86.7)	28/33. **84.9** (68.1–94.9)	28/33. **84.9** (68.1–94.9)	0.0016	45/54. **83.3** (70.7–92.1)	35/54 **64.8** (50.6–77.3)	0/54 **100** (93.4–100)	34/54 **63.0** (48.7–75.7)	34/54 **63.0** (48.7–75.7)	<0.0001
**HIV-uninfected**	17/20 **85.0** (62.1–96.8)	19/20 **95.0** (75.1–99.9)	16/20 **80** (56.3–94.3)	20/20 **100** (83.2–100)	19/20 **95.0** (73.1–99.8)	19/20 **95.0** (73.1–99.8)	20/20 **100** (83.2–100)	0.1111	16/12 **57.1** (37.2–75.5)	7/28 **25.0** (10.7–44.9)	0/28 **100** (79.4–100)	6/28 **21.4** (8.3–41.0)	6/28 **21.4** (8.3–41.0)	*<0.0001*
**Total**	34/53 **64.2** (49.8–76.9)	41/53 **77.4** (63.8–87.7)	31/53 **58.5** (44.1–71.9)	44/53 **83.0** (70.2–91.9)	43/53 **81.1** (68.0–90.6)	47/53 **88.7** (77.0–95.7)	48/53 **90.6** (79.4–96.9)	0.0002	61/82 **74.4** (63.3–82.4)	42–82 **51.2** (39.9–62.4)	0/82 **100** (88.8–100)	40/82 **48.8** (37.6–60.1)	40/82 **48.8** (37.6–60.1)	*<0.0001*

Footnotes: HIV: human immunodeficiency virus; TB: tuberculosis; TST: Tuberculin skin test; HCA: healthy community adults; QFT-GIT: QuantiFERON® Gold in Tube (indeterminate results included as negative results); SM: smear microscopy.

* X square test was used for statistical analysis.

The overall smear microscopy sensitivity evaluated in the PTB patients was 58.5% (**Table 8**); if we stratify for HIV status, smear microscopy sensitivity was significantly lower in the HIV-infected than the HIV-uninfected patients (p = 0.004). The overall specificity evaluated in HCA was 100%, independent of HIV status **(** **Table 8**).

#### Combination of smear with the TST and QFT-GIT

In 53 PTB patients concomitantly tested by the TST, QFT-GIT and smear microscopy, we further assessed whether the combination of tests improved their respective sensitivity and specificity for active TB diagnosis (**Table 8**). Among the PTB patients, the overall sensitivity was significantly different among the different tests when evaluated as single tests and combined tests (p = 0.0002). The difference was mainly due to the HIV-infected population in whom the sensitivity of the single tests increased significantly from below 60% up to 84.9% (0.0016) **(** **Table 8**). To note: the sensitivity of smear microscopy alone among the HIV-infected PTB patients was statistically different when compared to that combined with all 3 tests (QFT-GIT, TST and smear microscopy) (p = 0.0003) or with the IGRA and the microbiological test (QFT-GIT with smear microscopy) (p = 0.008) (**Tables 9** **and** **10**). These results indicate that in HIV-infected PTB patients the combination of microbiology tests with immunological tests increases the possibility of diagnosing active TB (**Table 10**).

**Table 9:** p values of multiple comparisons among tests evaluating sensitivity for active TB in active pulmonary TB patients (as reported in Table 6) in all patients with or without HIV-infection.

	Single test					Combined tests		
	TST	QFT-GIT	SM		TST+QFT-GIT	TST+SM	QFT-GIT+SM	QFT-GIT+SM+TST
**TST**	NA	0.20	0.69		0.04	0.08	**0.005** **	**0.0021** **
**QFT-GIT**	–	NA	0.06		0.63	0.81	0.20	0.11
**SM**	–	–	NA		0.009*	0.02	**0.0008** **	**0.0003** **
**TST+QFT-GIT**	–	–	–		–	–	–	0.39
**TST+SM**	–	–	–		–	–	–	0.26
**QFT-GIT+SM**	–	–	–		–	–	–	1

Footnote: HIV: human immunodeficiency virus; TB: tuberculosis; TST (Tuberculin skin test); QFT-GIT: QuantiFERON® Gold in Tube (indeterminate results included as negative results; SM: smear microscopy.

* Significant after Bonferroni correction for 5 multiple comparison, p value≤0.01;

** significant after Bonferroni correction for 7 multiple comparison, p value≤0.007.

**Table 10:** p values of multiple comparisons among tests evaluating sensitivity for active TB in active pulmonary TB patients (as reported in Table 9) with HIV-infection.

	Single test				Combined tests			
	TST	QFT-GIT	SM	TST+QFT-GIT		TST+SM	QFT-GIT+SM	QFT-GIT+SM+TST
**TST**	NA	0.32	0.81	0.12		0.12	**0.007** **	**0.007** **
**QFT-GIT**	–	NA	0.13	0.78		0.79	0.15	0.15
**SM**	–	–	NA	0.04		0.04	**0.002** **	**0.002** **
**TST+QFT-GIT**	–	–	–	–		–	–	0.36
**TST+SM**	–	–	–	–		–	–	0.37
**QFT-GIT+SM**	–	–	–	–		–	–	1.0

Footnote: HIV: human immunodeficiency virus; TB: tuberculosis; TST (Tuberculin skin test); QFT-GIT: QuantiFERON® Gold in Tube (indeterminate results included as negative results; SM: smear microscopy.

* Significant after Bonferroni correction for 5 multiple comparison, p value≤0.01;

** significant after Bonferroni correction for 7 multiple comparison, p value≤0.007.

## Discussion

We present the results of a prospective multicenter trial conducted in India (a country highly endemic for TB) that was designed to investigate the performance of a diagnostic toolbox for TB. Our previous report deals with the microbiological tests [8]. Using the same populations, immunological tests (QFT-GIT and TST) were performed as additional diagnostic tools for active TB and to evaluate their respective performances in populations of adults with varying LTBI risks.

Recently introduced for the diagnosis of LTBI, IGRAs have been evaluated in active-TB disease as an indirect marker for measuring its diagnostic value in varying populations from endemic and non-endemic countries [16]. As recently reviewed, IGRAs have very high specificity and are unaffected by prior BCG vaccination or sensitization to non-tuberculosis-mycobacteria (NTM) [6], [17]. Such assays might be mostly appropriate in India because a great majority of individuals have been vaccinated with BCG [18] and NTM infection is also highly prevalent [19]. By this study, we confirmed the data generated in India showing that QFT-GIT positivity is significantly associated with HIV-uninfected active-TB patients [20].

Our study reports that the overall sensitivity of QFT-GIT (77.4%) was higher than the TST sensitivity (63.2%) when the PTB patients were concurrently tested, but the difference was not significant (p = 0.42). The overall QFT-GIT sensitivity was slightly higher in the overall QFT-GIT tested group (82.3%) than in the group of patients concurrently tested with the TST (77.4%). The quality of the sub-grouping procedure was good: the QFT-GIT sensitivity in PTB patients with TST records was not significantly different from the overall group (p = 0.71) or those without TST records (p = 0.15), and was independent of the HIV status. The overall specificity for active TB of the QFT-GIT (51.2%) among the concurrently tested non-TB HCA was significantly lower compared to the TST (74.4%) (p = 0.04). The poor specificity of the QFT-GIT and TST obtained in this study was expected, due to the high proportion of LTBI in India [17]–[20].

However, the diagnostic values of both tests varied according to the HIV status. Our study reports that the overall “clinical” sensitivity of QFT-GIT (including the indeterminate results as negative) in PTB patients, was 82.3% and the “laboratory” sensitivity (after exclusion of indeterminate results) was 92.9%. Moreover, the “clinical” sensitivity of QFT-GIT was significantly lower in HIV-infected patients than in HIV-uninfected active-TB patients (p<0.0001). Similarly, the TST sensitivity among the PTB was significantly lower in the HIV-infected (51.5%) than the HIV-uninfected (85.0%) patients (p<0.0021). These results are in agreement with two recent Indian studies performed in HIV-infected PTB patients, pointing out that a proportion of positive results is significantly lower if the CD4 counts are smaller than 200 cells/mm^3^ [21], [22].

The lower QFT-GIT “clinical” sensitivity compared to the “laboratory” sensitivity in the HIV-infected PTB patients was totally associated with the high proportion of indeterminate results, which were significantly higher in this group compared to the HIV-uninfected individuals (p<0.0001). Subsequently, when indeterminate results were excluded, the “laboratory” sensitivity of IGRA was not significantly different among the HIV-uninfected (91.4%) and HIV-infected (89.3) PTB patients (p = 0.71). The proportion of indeterminate results in the HIV-infected PTB patients (23.7%) was similar to the rates observed in other studies in similar immunocompromised populations [21]–[23]. In our study, all the indeterminate results were due to the poor IFN-γ production in the positive control tube, which might be related to profound immunosuppression. In fact, in the literature, the percentage of indeterminate results varied and was inversely related to the average number of CD4 counts [22], [23]. However, in our study only a limited number of HIV-infected TB patients were recently tested for CD4 counts and therefore no correlation could be made. The rate of indeterminate results in HIV-uninfected PTB patients was only 3.5%, similar to the rates observed in other studies [17], and was higher compared to the HIV-uninfected HCA (0.0%), but the difference was not significant (p = 0.49).

Despite its well-known limitations with respect to accuracy and reliability, the TST is still widely used in initial screenings for LTBI in developed countries, and was shown to be useful in studies evaluating the IGRAs in active TB [24]. In this meta-analysis, the TST sensitivity ranged from 66% to 100%, with a pooled estimate of 77%; its specificity was consistently high, with a pooled estimate of 97% in non-BCG-vaccinated populations. However, its specificity was lower in BCG-vaccinated populations and highly heterogeneous, ranging from 35% to 79%. In our study, the overall TST sensitivity among the PTB patients was 64.2% with a specificity of 74.4%. This low TST specificity might be associated with the high coverage of BCG vaccination in the Indian population [18] and/or with Non-Tuberculosis Mycobacteria (NTM) sensitization [19]. However, as pointed out by Farhat and coll., NTM is not a clinically important cause of false-positive TST, except in populations with a high prevalence of NTM sensitization and a very low prevalence of TB infection [25], which is not the case in our population in India.

Furthermore, the TST diagnostic values varied according to the population studied. For instance, a significant lower sensitivity was shown in the HIV-infected (51.5%) than in the HIV-uninfected (85.0%) PTB patients (p<0.0021). Using a cut-off point of 5 mm, the sensitivity slightly increased in the former (51.5% to 54.1%), but the difference still remained not significant (p = 1). Likewise, among both PTB patients and HCA, the frequency of complete unresponsiveness of the TST was significantly higher in the HIV-infected than in the HIV-uninfected individuals. This is in line with the results of a cohort of HIV-infected patients published by Elzi and collaborators [26] showing that the TST response was either a present or absent system (“ON-OFF”). As reported elsewhere [27], the low TST response in HIV-infected active-TB patients was linked to a more profound immunosuppression associated with mycobacterial dissemination, probably related to the T-regulatory cells at the sites of the TST [28]. However, due to the relatively low number of patients having a recent CD4+ T-cell count, the statistical power was not evaluated in this study.

As already shown by Catanzaro and colleagues [29], the varying degree of a physician’s CSTB has a definite influence on the accuracy clinical symptoms, radiological findings and microbiological tools and was confirmed in our preceding study [8]. In contrast, in our present study the varying degree of a physician’s clinical suspicion of TB has no influence on the accuracy of the QFT-GIT. These results might be associated with the absence of influence of duration and severity of the disease on the T -cell immune responses. Our study also gave evidence that the QFT-GIT results in PTB patients were not associated with the sputum mycobacterial yield (smear status and grade, liquid culture status, TTP of culture) as recently reported by Theron [30]. By contrast, a smaller study of HIV-infected TB patients in a similar setting found a significant but weak correlation between quantitative T-SPOT.TB responses and smear grade (a relatively crude measure of bacterial load), but none with TTP culture [31]. As already described, our study showed that the recently cured TB patients still respond to the QFT-GIT (91.7%) after 2 years of a successful therapy. Therefore, this assay cannot be used to monitor TB therapy efficacy [32], contrary to experimental IGRAs based on RD1 selected peptides [12], [32], [33].

Due to the smear-status independence upon immunological tests, we combined the microbiological tests with the QFT-GIT and TST. Interestingly, compared to smear microscopy alone, the combination of immunological tests (QFT-GIT, TST) with smear microscopy significantly increased the sensitivity for PTB diagnosis (p = 0.003), especially in the HIV-infected patients (p = 0.002). This result indicates that the immune tests may be important supporting tools that can increase the detection rate of active-PTB in HIV-infected patients.

In conclusion, in this study conducted in India, a high TB burden country, we show that the QFT-GIT and TST have similar accuracy for active-PTB diagnosis. Moreover, in HIV-infected patients, a combination of smear microscopy with both immunological tests significantly increases the sensitivity for active disease diagnosis compared to smear microscopy alone.
